# Don’t Discount Societal Value in Cost-Effectiveness: Comment on "Priority Setting for Universal Health Coverage: We Need Evidence-Informed Deliberative Processes, Not Just More Evidence on Cost-Effectiveness"

**DOI:** 10.15171/ijhpm.2017.03

**Published:** 2017-01-14

**Authors:** William Hall

**Affiliations:** Centre for Clinical Epidemiology & Evaluation, Vancouver Coastal Health Research Institute, Vancouver, BC, Canada.

**Keywords:** Cost-Effectiveness, Priority Setting, Resource Allocation, Multi-Criterion Decision Analysis (MCDA), Deliberative Processes

## Abstract

As healthcare resources become increasingly scarce due to growing demand and stagnating budgets, the need for effective priority setting and resource allocation will become ever more critical to providing sustainable care to patients. While societal values should certainly play a part in guiding these processes, the methodology used to capture these values need not necessarily be limited to multi-criterion decision analysis (MCDA)-based processes including ‘evidence-informed deliberative processes.’ However, if decision-makers intend to not only incorporates the values of the public they serve into decisions but have the decisions enacted as well, consideration should be given to more direct involvement of stakeholders. Based on the examples provided by Baltussen et al, MCDA-based processes like ‘evidence-informed deliberative processes’ could be one way of achieving this laudable goal.

## Introduction

As healthcare organizations around the world face a scarcity of resources to fund growing demand, the need to set priorities to guide allocation is becoming increasingly critical. To support this effort, Baltussen et al paper provides an in-depth discussion of the processes and criteria that should be used to guide priority setting. More specifically, they argue that restricting criteria to cost-effectiveness does not respect the inherently political nature of priority setting itself. As an alternative to using cost-effectiveness exclusively, Baltussen et al propose using ‘evidence-informed deliberative processes’ to guide the criteria development and priority setting process. Multiple examples of application are provided including HIV Control in Indonesia, and Reimbursement Decisions in Health Technology Agencies.^1^ In this editorial, a re-examination of Baltussen et al central thesis will be presented along with additional commentary related to the use of ‘evidence-informed deliberative processes.’

To begin, a point of clarification: in Baltussen et al paper, the term cost-effectiveness analysis (CEA) appears to be used to describe analyses that could include both natural units (eg, cancers detected, reduction in blood pressure, falls avoided) and utilities in terms of ‘healthy years’ as measures of benefit.^1,2^ This covers both the conventional definition of CEA as well as cost utility analysis (CUA).^2^ Both types of analyses are based in an extra-welfarist paradigm whereby the benefits and costs derived from an intervention are compared against a control or current standard of care.^2-4^ Quality adjusted life years (QALYs) have been the widely accepted reference standard in CUA, and serve as a measurement of health.^2,5^ Using an incremental cost-effectiveness planes, decisions are made as to whether a new intervention should be adopted over the current standard of care.^2^ Standard units (QALYs) allow for comparability between distinct interventions, and facilitate the allocation of resources based on these results.^2^ In this way, ‘health’ could be maximized by funding interventions that deliver the greatest value (ie, largest benefit with the smallest cost) as determined by the results of these analyses.

## Societal Values

In their argument against the sole use of CEA to guide the allocation of resources, Baltussen et al posit that priority setting is a “value laden political process, in which multiple criteria beyond cost-effectiveness [ie, health or QALY maximization] are important.” Social values including “caring for the worse off in society or responsibility for one’s own health” are provided as examples.^1^ While they claim that this may call for a “paradigm shift in how research should approach the challenge of priority setting,” the notion that a singular measure of benefit is insufficient to fully capture the benefits of an intervention has been – and continues to be – grappled with in the realm of CEA methodology.

An early example of this desire to broaden the perspective of resource allocation decisions was described by Sen who argued that a focus on individual utility via welfare economics was too narrow, and “ought to be replaced by an approach that took the quality of utility and peoples’ capabilities into account.”^3,6^ Indeed, this rejection of an exclusive focus on individual utility and broadening of the evaluative space to include other desiderata including ‘health’ was one of the “seeds” from which extra-welfarism and CEA has grown.^3^ Further, an acknowledgement of health as a ‘merit good’ or ‘basic good’ and the need to distribute it equally within a society forms another tenet of extra-welfarism.^7-9^ Since the founding of these tenets of extra-welfarism, developments of CEA methodology to better capture the benefits of healthcare interventions – and reflect the values of a society – have been created including: multiple instruments to measure health, weighted adjustments, and broader measurement of cost.

While the EQ-5D and SF-6D are two of the traditional approaches used to measure QALYs in patients receiving interventions, factors including well-being of the patient and impact on patients’ families have been incorporated into newer instruments to allow for a broader conception of health.^10-12^ Additional measures of utility have also been recognized and included in analyses including the experience of patients undergoing interventions ie, their process utility.^13,14^ Weighted adjustments of health measurements have also been proposed and carried out to more accurately reflect the benefits of treatments with respect to a society’s preferences. These adjustments have included weighting benefit with respect to age and equity. For example, attributing more weight to benefits experienced by younger patients,^15^ to older patients,^16^ to patients of working age,^17^ to those with poor levels of initial health, to those that stand to lose a larger proportion of their health, or to those that have a lower socioeconomic status.^16,18,19^ While these approaches have focused on redistributing the benefits of interventions, attempts to reflect societal values have also been addressed when accounting for costs of interventions. For example, the inclusion of indirect costs to a society in order to reflect the productive contribution of persons at different ages.^20^ In this way, societal values and factors could and have been quantified and incorporated into CEA to better capture the benefits of interventions and reflect societal preferences.^21^

While this methodology is certainly not perfect (and perhaps never will be), the aforementioned areas of research would counter Baltussen et al argument that CEA cannot accommodate social values into its calculations. One could argue that these methods could even provide a more rigorous measurement and analysis of societal values than ‘evidence-informed deliberative processes’ which rely more heavily on expert opinion during “deliberative discussion among consultation panel members … to reach agreement on the final rank order of interventions.”^1^ In fact, this concentration of power in the hands of a select group on behalf of a population has been a criticism of multi-criterion decision analysis (MCDA) approaches like ‘evidence-informed deliberative processes.’^22^

If it is accepted that CEA methodology has some ability to capture societal values in its measurement and analysis, then this distinction between CEA and ‘evidence-informed deliberative processes’ (and all other MCDA processes) lies in the methods used to quantify these values – and not in an ability or inability to do so.

## Stakeholder Engagement

While Baltussen et al first argument that ‘evidence-informed deliberative processes’ are able to capture societal values (while CEA methodology cannot) necessitates further discussion, their second argument surrounding stakeholder involvement in these processes is very compelling from both a theoretical and practical standpoint.

Based on the theoretical foundation provided by ‘Procedural Justice’ whereby those individuals affected should have the opportunity to participate in the decision-making process, Baltussen et al cite the work of Daniels and Sabin to support their assertion that stakeholders are “more likely to confer legitimacy to [a] decision” that emanates from a process that has included them meaningfully.^1,23^ Theoretically, this argument is also supported by ‘values-based evaluation’ that follows social constructivist ontologies and suggests that broader social impacts should be included by considering all relevant perspectives in an evaluation.^24-26^ Engagement from staff and the public has also been identified as an element of high performance in priority setting and resource allocation process, and as facilitator to effective resource allocation in other MCDA processes.^27,28^

While CEA methodology might be able to account for societal values (or any other values that might be deemed relevant by decision-makers), the collection and analysis of data as well as the recommendations delivered to decision-makers will likely be conducted by investigators or scientists rather than stakeholders. Although this may allow for more rigorous methods of valuation, the results may encounter greater resistance since the stakeholders who are impacted by the changes were not directly involved in the process.

## Ethical issues

Not applicable.

## Competing interests

Author declares that he has no competing interests.

## Author’s contribution

WH is the single author of the paper.
